# Resorbable optical fibers for interstitial photodynamic therapy—assessment of photosensitizer spatial distribution in tumors

**DOI:** 10.1117/1.JBO.30.5.058001

**Published:** 2025-05-14

**Authors:** Jawad T. Pandayil, Stefan Šušnjar, Muhammad Daniyal Ghauri, Sanathana Konugolu Venkata Sekar, Johannes Swartling, Davide Janner, Nadia G. Boetti, Nina Reistad

**Affiliations:** a Fondazione LINKS-Leading Innovation and Knowledge for Society, Torino, Italy; b Politecnico di Torino, Dipartimento di Scienza Applicata e Tecnologia (DISAT) and RU INSTM, Torino, Italy; c Lund University, Department of Physics, Lund, Sweden; d SpectraCure AB, Lund, Sweden; e Tyndall National Institute, Cork, Ireland; f BioPixS Ltd– Biophotonics Standards, IPIC, Cork, Ireland

**Keywords:** photodynamic therapy, bioresorbable photonics, photosensitizer distribution, optical fibers, diffuse optical tomography, fluorescence

## Abstract

**Significance:**

Optical-quality bioresorbable implants, which gradually dissolve within the body, are gaining increasing interest due to their potential to eliminate the need for revision surgeries. These implants show significant promise in treating deep-seated tumors in high-risk areas, such as the brain, and offer extended capabilities for monitoring interstitial physiological parameters or pharmacokinetics through photonic technologies.

**Aim:**

A proof-of-principle validation has been conducted on calcium phosphate glass (CPG)-based bioresorbable optical fibers to assess their capability to monitor the spatial distribution of photosensitizing (PS) drugs in tumors—an essential parameter to optimize for enhanced treatment outcomes in photodynamic therapy (PDT).

**Approach:**

*Ex vivo* validation was performed on liquid phantoms with solid tumor-mimicking inclusions containing the fluorescent PS drug. In-house developed bioresorbable fibers, with optical characteristics similar to silica fibers used in current PDT systems, were utilized. For the first time, these fibers were used for the interstitial acquisition of fluorescent signals, followed by the tomographic reconstruction of the drug distribution in the phantom. The results were compared with those obtained from a standard clinical system equipped with silica fibers.

**Results:**

The reconstructed drug distribution with bioresorbable fibers agreed with that obtained using the same system with standard silica fibers.

**Conclusions:**

We reveal the potential of further exploring CPG bioresorbable optical fibers for interstitial PDT.

## 1 Introduction

Photodynamic therapy (PDT) is a promising cancer treatment modality that involves administering a photosensitizing (PS) drug either topically or intravenously, followed by irradiation of the light at a wavelength that aligns with the PS’s absorbance band. In the presence of oxygen, this initiates a cascade of events leading to direct tumor cell death, damage to the tumor’s microvasculature, and the activation of a localized inflammatory response.^1^^–^^3^ The effectiveness of PDT depends on a combination of parameters^2^^,^^4^ including the type and dose of the PS, the timing between its administration and light exposure, knowledge of the distribution of PS within the tumor, tumor oxygen levels, total light dose, and fluence rate and PDT-induced blood flow changes.^5^^,^^6^ There is a growing interest in developing integrated systems that allow real-time interstitial monitoring of these parameters during PDT.^7^^–^^9^ These systems are designed to enable real-time fluence modulation, delivering a more uniform dose to the tumor and improving treatment precision and outcomes. Such advancements are expected to enhance the performance of current clinical interstitial PDT systems, which remain underutilized in clinical practice despite being the first Food and Drug Administration (FDA)-approved drug-device combination in the 1990s.^10^

Fiber optic technology is widely used for endoscopic and interstitial light delivery in PDT, enabling the treatment of deep-seated tumors and internal organs.^11^ In clinical practice, silica-based fiber-optic endoscopy is a well-established method for guiding light. Nevertheless, these endoscopes are inserted only during the procedure and are not suitable for extended monitoring.^12^ A major limitation of traditional silica-based fibers is their non-degradable nature, requiring surgical removal after treatment. This not only increases the risk of infection, fibrosis, and procedural complications but also poses a significant challenge in delicate, high-risk environments such as the brain.^13^^–^^15^ In multi-session PDT, the repeated insertion and removal of fibers exacerbate patient discomfort, procedural risks, and clinical workload.^14^

The motivation for this study stems from the clinical need for precise, minimally invasive, and self-dissolving light delivery and real-time monitoring systems for interstitial PDT, particularly for the treatment of deep-seated and surgically inoperable tumors such as glioblastoma, head and neck, or intra-abdominal tumors.^16^^,^^17^ An ideal interstitial PDT tool for such scenarios should efficiently deliver light to deep tumor tissues while naturally degrading post-treatment, eliminating the need for removal, enabling real-time dosimetry to ensure uniform and effective light exposure, and allowing minimally invasive monitoring of drug distribution to optimize therapeutic outcomes. With the development of novel biocompatible and degradable optical fibers for interstitial PDT applications, new therapeutic tools are becoming possible.^18^^,^^19^ Relevantly, bioresorbable optical fibers based on calcium phosphate glass (CPG) meet these criteria by combining bioresorbability, optical efficiency, and functional integration.^20^ Their composition mimics bone minerals, ensuring biocompatibility. They dissolve in physiological media over a clinically relevant time, bypassing the necessity of extraction procedures.^21^^,^^22^ Their optical properties can be tailored based on the composition. To the best of our knowledge, CPG fibers possess the lowest optical loss in the category of biocompatible and degradable optical fibers reported in the literature.^23^ Along with light delivery, they possess the potential for extended and real-time monitoring of relevant physiological signals, tissue characteristics, or biochemical information.^24^

CPG-based low-loss optical fibers were first developed using a fiber drawing technique in 2016 by Ceci-Ginistrelli et al.^20^ Since then, they have been extensively studied for their *in vitro* solubility,^25^ biocompatibility, and mechanical properties.^26^ Systematic investigations into their degradation behavior have demonstrated complete dissolution within 1 month, with less than 10% variation in optical power transmission during the first 2 weeks of fiber dissolution.^25^ In a study by Podrazký et al.,^27^ mice subcutaneously implanted with CPG fibers showed no clinical signs of adverse effects for up to 5 weeks. CPG fibers possess good thermomechanical properties and have also been recently validated for diffuse optical techniques such as diffuse optical spectroscopy (DOS)^28^ and diffuse correlation spectroscopy (DCS)^29^ for monitoring complementary physiological parameters, paving the way for interstitial continuous monitoring of tissue oxygenation, hemodynamics, and metabolism. Despite their benefits, CPG fibers have not been explored for interstitial PDT application.

This study investigates the potential of CPG fibers for light delivery and monitoring in interstitial PDT. Specifically, our objective was to assess whether these fibers could be used to accurately map the spatial distribution of PS drugs within tumor-mimicking solid phantom inclusions using diffuse fluorescence tomography (DFT). We utilized custom-fabricated multi-mode (MM) CPG fibers along with a well-tested solid-liquid phantom approach.^30^ The performance of CPG fibers was evaluated against standard silica fibers in terms of their ability to map the PS distribution within the phantoms. This novel approach represents a step toward a minimally invasive therapeutic device that would reduce patient burden and improve the treatment efficacy of deep-seated tumors.

## 2 Materials and Methods

### 2.1 CPG Fiber Fabrication

MM bioresorbable CPG optical fiber employed in this study was fabricated through a rod-in-tube technique using an in-house developed drawing tower.^20^ The glasses for the fiber were made by conventional melt quenching method using high-purity biocompatible chemicals ($P_{2}O_{5}\cdot \mathrm{CaO}\cdot {\mathrm{Na}}_{2}O\cdot {\mathrm{SiO}}_{2}\cdot \mathrm{MgO}$). The wavelength-dependent refractive index n(λ) of the core ($n_{\text{core}}$) and cladding ($n_{\text{cladd}}$) glasses were then measured at different wavelengths (633, 825, 1061, 1312, and 1533 nm) using a Metricon 2010 prism coupler (Metricon Corporation, United States), with an estimated error of ±0.0005. The refractive index values at the wavelength 690 nm (i.e., the wavelength used in DFT experiments reported here, see Sec. 2.2) were then obtained through interpolation using Cauchy’s equation. These values were $n_{\text{core}}=1.5323$ and $n_{\text{cladd}}=1.5135$. The refractive index difference in core and cladding glass was attained through increasing MgO molar content in the core glass as a substitute for CaO, as detailed in the work by Ceci-Ginistrelli et al.^20^ The numerical aperture (NA) of the CPG fiber was calculated as $\mathrm{NA}=\sqrt{n_{\text{core}}^{2}-n_{\text{cladd}}^{2}}=0.24$. This value of NA for CPG fibers is close to the NA=0.22 of standard silica fibers (Spc Sterile Bare Fiber UVH 400 N SC, LightGuideOptics International Ltd., Germany) employed in clinical PDT systems.

The preform of the fiber consists of a core rod obtained by casting the melt into a 12-mm-diameter cylindrical mold, and a cladding tube with a 10.7-mm outer diameter, realized through an in-house extrusion facility.^31^ The core rod was then stretched to 5.4-mm diameter to fit into the cladding tube. The stretched rod was inserted in the cladding tube and the rod-in-tube preform was further stretched into several 1-m fiber sections using the fiber drawing tower. Optical microscopy (Nikon ECLIPSE E 50i, Nikon Instruments Inc., United States) equipped with the image analysis software ToupView (ToupTek Photonics, China) was used to measure the fiber diameter at the fiber’s cross-section. The measured core diameter of the CPG fiber closely matches the diameter of the standard silica fiber (400  μm). The details about the fabrication can be found in previous publications.^20^^,^^31^

For practical experimental convenience, the fabricated fibers were cleaved (CT-106, Fujikura, Japan) into 11 sections of 25 cm each (see Sec. 2.3), which were SMA connectorized at one end by following the protocol for the connectorization of optical fibers.^32^

### 2.2 Phantom Preparation

Tissue-mimicking phantoms with tumorous inclusions were realized. A well-tested hybrid solid-liquid phantom approach based on our previously reported work was followed.^30^ Briefly, the gelatin-based solid inclusion phantom representing the tumor was submerged in a liquid intralipid-based phantom. Two inclusions, one spherical (volume of $0.6\;{\mathrm{cm}}^{3}$) and one ellipsoidal (volume of $3.2\;{\mathrm{cm}}^{3}$), were made and stored for later use. These inclusions contained fluorescent photosensitizer verteporfin, an active substance of the PS drug Visudyne (Cheplapharm Arzneimittel GmbH, Germany). A 0.67  mg/kg fluorophore concentration (in milligrams) per phantom mass (in kilograms) was maintained in both inclusions. Following the referenced work by Ghauri et al.,^30^ the optical properties targeted for the solid phantom inclusion (mixture of gelatin, water, intralipid, and India ink) at 690 nm were absorption $\mu_{ai}=0.20\;{\mathrm{cm}}^{-1}$ and reduced scattering coefficient ${\mu_{si}}^{\prime }=15.7\;{\mathrm{cm}}^{-1}$, mimicking the optical properties of the tumorous region.

Intralipid-based liquid phantom, mimicking surrounding tissue, was prepared by mixing 4.9 ml of 1% India ink solution (Rotring, Germany) and 35.4 ml of intralipid 20% (Fresenius Kabi, Ltd., Germany) in 960 ml of purified water in a glass beaker, which was placed on a magnetic stirrer for mixing. The recipe was chosen to have the optical properties of absorption $\mu_{abg}=0.30\;{\mathrm{cm}}^{-1}$ and reduced scattering ${\mu_{sbg}}^{\prime }=8.3\;{\mathrm{cm}}^{-1}$ at 690 nm. Each of the gelatin phantoms stored in the freezer at −18°C was taken out and kept for a sufficient time (around 45 min) at room temperature, to bring it toward a thermodynamic equilibrium with the environment and attain mechanical properties convenient for inserting the fibers within inclusion during the measurement (see Sec. 2.3).

### 2.3 Experimental Setup and Measurements

**Fig. 1:**
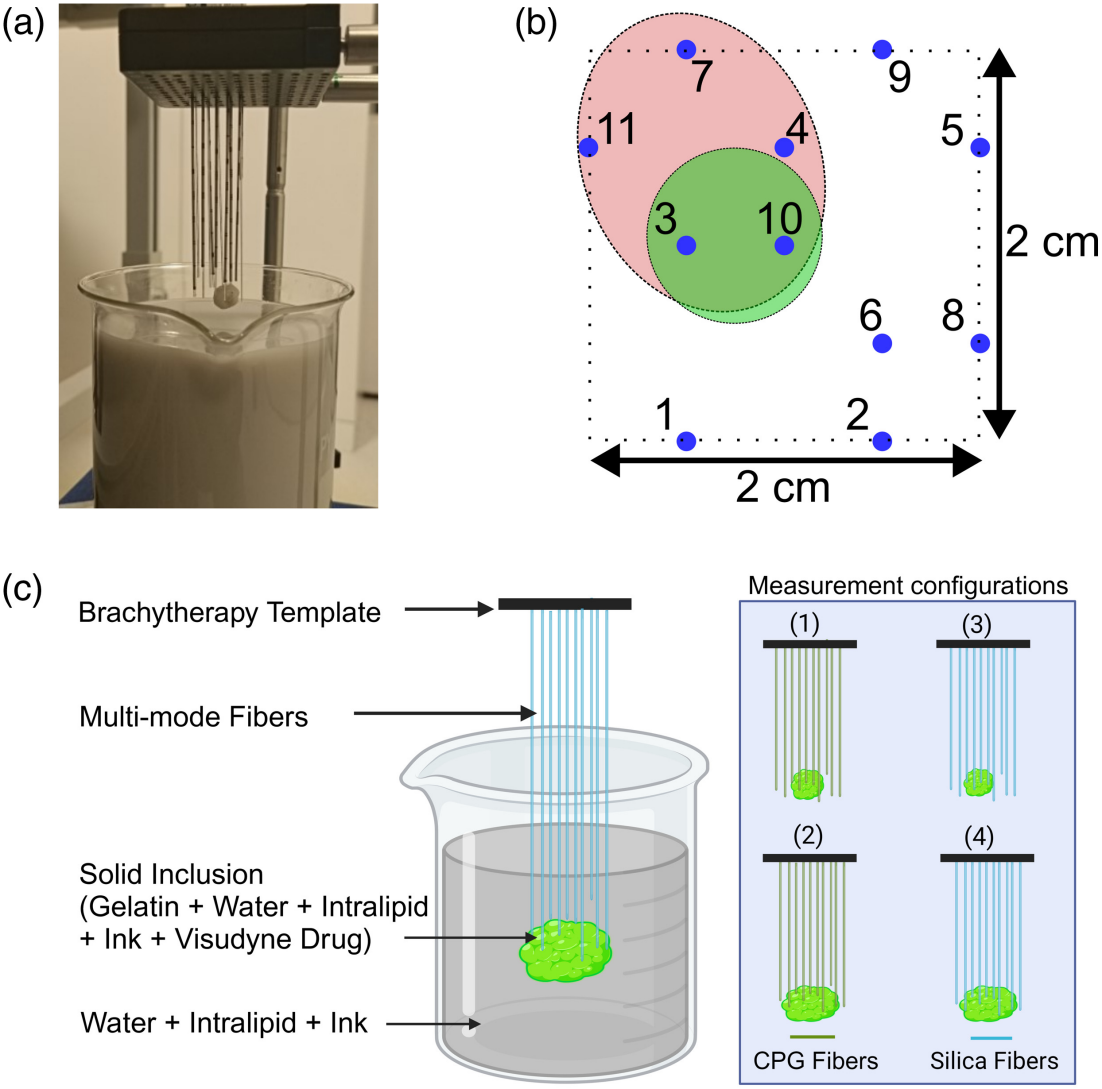
(a) Experimental setup with CPG fibers, liquid tissue-mimicking phantom, and solid gelatin-based tumor-mimicking fluorescent inclusion, before the measurement. (b) Schematic showing the fiber positioning within the inclusions—spherical (green) and ellipsoidal (red)—where the blue markings represent the 11 fiber centers in the plane (horizontal) parallel to the brachytherapy template. (c) Schematic representing the hybrid phantom and fibers assembly during the measurement (left), along with the simplified schematics of all four measurement configurations (right).

The phantoms and fiber assembly is shown in Fig. 1(a). Two sets of 11 optical fibers each, one with CPG, and the other with standard silica fibers, were used for the measurements. All 11 CPG fibers from the first set were inserted through a fixed brachytherapy template and positioned within 2  cm×2  cm area of the template [Fig. 1(b)]. This number of fibers was chosen to have enough spatial sensitivity to reconstruct the inclusion and background^33^ while not complicating the setup with a dense distribution of fibers.

The glass beaker of 10 cm inner diameter, containing 1 l of the liquid phantom, was placed on a lab jack, under the brachytherapy template. Gelatin-based tumor-mimicking spherical solid inclusion was fixed at the CPG fiber tips [see Fig. 1(c)], in the first measurement configuration. The lab jack was vertically adjusted so that the center of the solid tumor-mimicking inclusion was immersed in the liquid at a depth of 7.5 cm from the surface, and height of 5.5 cm from the bottom [Fig. 1(c)]. In the second measurement configuration, the spherical inclusion was replaced by the ellipsoidal one, within the same liquid phantom. In the case of the spherical inclusion, two fiber tips were within the inclusion, whereas in the case of the ellipsoidal, five fiber tips were within the inclusion [see Fig. 1(b)].

The same experimental procedure was then repeated with the set of silica fibers instead of CPG fibers, inserted at the same 11 positions in the brachytherapy template as before. The same liquid phantom, and the same solid inclusions as in the first two measurement configurations were used. The spherical one was used in the third configuration and placed at the same position as in the first, whereas the ellipsoidal one was used in the fourth configuration and placed at the same position as in the second [Fig. 1(c)].

During the measurements, PDT-like light delivery was conducted using SpectraCure’s P18 system comprising 18 photonics modules (PMs) to which the optical fibers can be connected. Each PM is capable of delivering light at 690 nm (Laser diode LDX-2405-690, LDX Optronics Inc., United States) and detecting (Si photodiode S12915-5K, Hamamatsu Photonics, Japan) both light around that excitation wavelength as well as fluorescent light at longer wavelengths (>700  nm). Two-meter-long silica fibers and 25-cm-long CPG fibers (SMA connectorized) were connected to SC ports in the P18 system via 45-cm SC-SMA patch cables. The choice of this rationale for using shorter sections of CPG fibers is based on the possible future clinical implantation, where long fibers are not required.^34^ The PMs were calibrated to deliver 170 mW at the output of the SC-SMA patch cable, in accordance with the system specifications.

A complete measurement consists of five cycles. Each measurement cycle involves continuous light delivery from one fiber during a particular time interval while all the other fibers detect this excitation and fluorescence emission light. The cycle is continued by the other fibers sequentially delivering light, one after another (see Table 1). The light delivery from each fiber lasted ∼3  s followed by a delay of 0.1 s for shifting the light delivery to the next fiber. The 3 s light delivery per fiber was long enough to reliably measure excitation and fluorescent light without causing photobleaching of the PS due to longer exposure time.^30^ The measurement data points are obtained by taking the average from all five cycles. Before each measurement, the setup was covered with a dark, light-proof curtain. The summary of all the measurements is given in Table 1.

**Table 1:** Details of the measurements conducted including the types of fibers used, the shape of the inclusion, and the protocol followed during each acquisition cycle.

	Fiber type	Inclusion shape	Protocol in one measurement cycle
1	CPG	Sphere	Light delivery from PM1 through fiber 1 and simultaneous light collection by fibers 2–11 (∼3 s). Switching the light delivery sequentially from PM1 to PM11, with subsequent light collection by all other fibers before each switch. Acquisition of 11×10=110 data points.
2		Ellipsoid	
3	Silica	Sphere	
4		Ellipsoid	


### 2.4 Data Processing and Reconstruction

Data collected from tomographic measurements were first processed such that the fluorescent signals below the defined threshold $P_{th}=5.4\cdot 10^{-11}\;W$ were set to zero, and all other fluorescent signals were reduced by the same threshold value $P_{th}$. This was done to remove the effect from unreliable data points (where the signal is too low compared with the measurement uncertainties), in a way similar to our previous work.^33^ The tomographic reconstruction algorithm used here was reported previously.^33^ Briefly, 110 data points (all source-detector fiber pairs from 11 fibers), represented as Born ratios between the detected power of fluorescent light (wavelengths >700  nm) and the detected power at the excitation wavelength (690 nm), were given as input to the reconstruction algorithm. The inhomogeneous medium of $(5.0\times 5.0\times 3.8)\;{\mathrm{cm}}^{3}$ was divided into a finite element mesh of $N_{e}=97,906$ tetrahedrons with $N_{n}=21,616$ nodes. The computer implementation was done using MATLAB (The Mathworks Inc., 2022) and the NIRFAST package.^35^^,^^36^ The reconstruction consists of calculating the forward model for every source-detector pair and solving the inverse problem by iteratively updating the estimates for the fluorescent yields in mesh nodes to minimize the discrepancy between the forward model vector F and the measurement data vector M. For a given vector of fluorescent yields in all nodes $\eta =(\eta_{1},\eta_{2},\ldots ,\eta_{N_{n}})$ and for a specific source-detector pair (s,d), the Born ratio from the forward model is^1^^,^^33^

$$F_{s,d}(\eta )=\frac{1}{G_{x}({\overset{\to }{r}}_{d},{\overset{\to }{r}}_{s})}\overset{N_{n}}{\underset{i=1}{\sum }}G_{x}({\overset{\to }{r}}_{i},{\overset{\to }{r}}_{s})G_{m}({\overset{\to }{r}}_{i},{\overset{\to }{r}}_{d})\eta_{i}\Delta V,$$ (1)

where ΔV is the element volume, $\eta_{i}$ is the fluorescent yield in node i (at the position ${\vec{r}}_{i}$), ${\vec{r}}_{s}$ and ${\vec{r}}_{d}$ are positions of the source and the detector, respectively, and $G_{x}$ and $G_{m}$ are Green’s functions solutions at the excitation and fluorescent emission wavelengths, respectively.^33^

The inverse problem is solved in two stages, referred to as S1 and S2. In S1, because the problem is ill-posed (the number of unknowns (nodes) is much greater than the number of measurement data points), the goal is to minimize the Tikhonov regularization cost function^1^^,^^33^

$$\Omega (\eta )={\left\|M-F(\eta )\right\|}^{2}+\lambda {\left\|L(\eta -\eta_{0})\right\|}^{2},$$ (2)

where λ is the regularization parameter, L is the regularization matrix, $\eta_{0}$ is the initial estimate for the vector of unknowns η, and ‖x‖ denotes Euclidean two-norm of vector x. The modification of the Levenberg-Marquardt algorithm was used to calculate the updates for fluorescent yield estimates according to^33^

$$\eta_{i+1}=\eta_{i}+(J_{i}^{T}J_{i}+\lambda_{i}L^{T}L{)}^{-1}J_{i}^{T}\delta (\eta_{i}),$$ (3)

where indices i and i+1 denote the ordinal number of the iteration and matrix $J=\frac{\partial F(\eta )}{\partial \eta }$ is the Jacobian of the system. The regularization parameter λ is initialized to the maximum of the diagonal of the Hessian matrix: $\lambda_{0}=\max\{\mathrm{diag}(J_{0}^{T}J_{0})\}$ and updated in every iteration to $\lambda_{i}=\max\{\mathrm{diag}(J_{i}^{T}J_{i})\}\cdot 10^{-i/4}$. The regularization matrix L was set to an identity matrix, so as not to bias the solution with *a priori* information about the geometry. The reconstructed fluorescent yield η is transformed to the fluorophore absorption $\mu_{af}$ using the relation $\eta =\gamma \mu_{af}$ and assuming homogeneous quantum efficiency of γ=10%,^37^^,^^38^ for fluorescence emission by verteporfin.

S2 of the reconstruction is described in our previous work^33^ and assumes the number of unknowns (originally the number of nodes $N_{n}$) is reduced to the number of regions ($N_{r}$) with homogeneous fluorescent yield. The regions are defined according to the results from S1. The introduction of S2 improves the accuracy of the solution with respect to S1,^33^ which is necessary for the quantitative performance comparison between CPG and standard silica fibers. The fluorescent yield estimate updates in S2 are still calculated according to Eq. (3), but without the regularization and with the reduced size of the Jacobian and the vector of unknowns.^33^ The iterative method stops when the relative decrease of the two-norm of the difference between the forward model and the measurements vector is <2%, or the maximal number of iterations is reached.^36^

## 3 Results

**Fig. 2:**
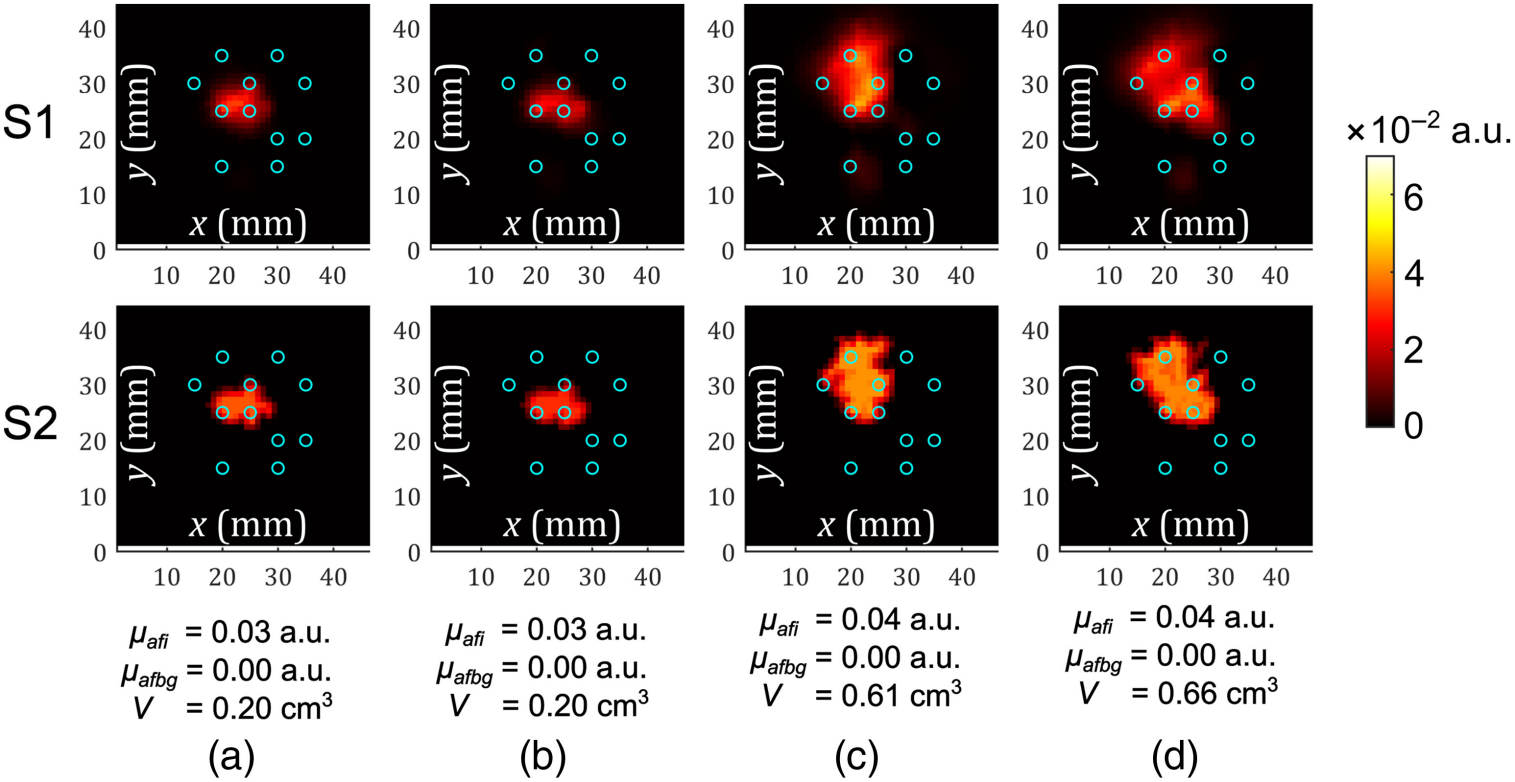
Tomographic reconstruction results for the measurements with (a) spherical inclusion and silica fibers, (b) spherical inclusion and CPG fibers, (c) ellipsoidal inclusion and silica fibers, and (d) ellipsoidal inclusion and CPG fibers. Slices through the planes cutting the inclusion in halves. Resulting fluorophore absorption (originating from PS) coefficients 2D color map in the first stage (S1) and in the second stage (S2) of the reconstruction shown in different rows. Numerical values for the reconstructed fluorophore absorption coefficients of the inclusion $\mu_{afi}$ and the background $\mu_{afbg}$, as well as the estimated volume of the inclusion V given below. Blue circles represent fiber projections in the xy-plane.

The results of DFT reconstruction from phantom measurements with silica and CPG fibers are shown in Fig. 2. The first two columns compare the performance of silica fibers (a) and CPG fibers (b) in retrieving the PS distribution in the phantom with the spherical tumor-mimicking inclusion. Columns (c) and (d) compare the performance of silica and CPG fibers, respectively, for the measurements with the ellipsoidal tumor-mimicking inclusion. The results shown in the first row (from above) are from S1, whereas the second row corresponds to the results from S2 (Sec. 2.4). The quantified fluorophore absorption and estimated volume of the inclusion obtained in S2 are also indicated in Fig. 2.

Considering the case of the spherical inclusion with the fluorescent PS drug inside, we do not observe any significant difference in the reconstructed fluorophore absorption values obtained using silica fibers and CPG fibers [Figs. 2(a) and 2(b)]. Similar can be said for the case of the ellipsoidal inclusion [Figs. 2(c) and 2(d)]. However, the reconstructed values for the fluorophore absorption coefficient are slightly different for spherical and ellipsoidal inclusions. The reconstructed volume is underestimated in all cases, for spherical and ellipsoidal inclusions. The true volume of the sphere was around $0.6\;{\mathrm{cm}}^{3}$ and of the ellipsoid around $3.2\;{\mathrm{cm}}^{3}$. The underestimation of the reconstructed volume leads to overestimating the reconstructed absorption,^33^ as discussed in Sec. 4. The reconstructed fluorophore absorption coefficients are more overestimated in the case of ellipsoidal inclusion compared with spherical inclusion because the volume of the reconstructed ellipsoid is more underestimated compared with the reconstructed sphere. When the reconstructed volumes are relatively close, as in Figs. 2(a) and 2(b), and Figs. 2(c) and 2(d), the reconstructed fluorophore absorption coefficients can be compared with each other.^33^ This implies that CPG and silica fibers performed similarly in DFT reconstructions from our measurements, demonstrating the potential of bioresorbable CPG fibers to monitor the distribution of PS drugs in PDT.

## 4 Discussion

The aim of this study was to determine the PS spatial distribution monitoring capabilities of CPG fibers within tissue-mimicking phantoms using DFT. The relative comparison between silica and CPG fibers showed good alignment in the reconstructed values for the fluorophore absorption coefficient and the volume of the tumor-mimicking inclusions. The two inclusions with different volumes and shapes were chosen to assess the potential of DFT for monitoring PS distribution regardless of tumor size and morphology. The absolute values for the volume are different from what was expected (the reconstructed volume is underestimated around 3 to 5 times with respect to the ground truth), hence also the absorption.

This volume underestimation was mainly due to the measurement data processing employed here, which is modified from our previous work.^33^ The present approach was chosen to trade off the optical power variation among CPG fibers. This power variation could have originated from the process of their in-house connectorization, where the bore size of the available standard SMA connectors (1040  μm) was greater than the diameter of the CPG fiber (870  μm). This might have caused a slight misalignment of the fiber from the central axis of the ferrule. As a consequence, along with CPG material losses,^20^ the power available at the fiber tip (in the tissue phantom) ranged 75 to 85 mW in different CPG fibers, compared with 150 mW obtained at the silica fiber tips. The variation in optical power among CPG fibers can be reduced by professional connectorization methods.

Because the use of the Born ratio (see Sec. 2.4) helps in canceling out many uncertainties regarding the fiber output and collected powers, there were no significant differences between the reconstructions with silica and CPG fibers. However, for a robust convergence to a solution of an inverse problem in all the considered cases (both with silica and CPG fibers), a certain level of signal corresponding to the estimated level of noise threshold (Sec. 2.4) had to be subtracted from every measurement data point, causing the volume underestimation.

As stated in our previous work,^33^ reconstruction results are sensitive to the true fiber positions, as well as the optical properties of the phantom. In our experiments, the information on the true fiber tip positions was not available once inserted into the phantom. Therefore, it was difficult to achieve exactly the same fiber and inclusion positioning in all four measurement configurations with different fiber types and inclusion shapes. Consequently, small quantitative differences between the reconstructions from the measurements with silica and CPG fibers were expected. It should be noted that the information on the fiber positioning as well as the estimated optical properties of tissue is already available in the clinical setting.^39^^,^^40^ Applying similar methods for estimating the implanted CPG fiber positions in future trials could pave the way for more reliable reconstruction.

Overall, in this first test of CPG fibers for DFT in PDT, we observed that there was no significant difference in the reconstruction results obtained from the tomographic measurements with silica and CPG fibers. It should be noted that this test model, comprised of SpectraCure’s P18 system for interstitial PDT and phantoms with PS verteporfin, was chosen because it was readily available for a proof-of-principle demonstration. Although the P18 system is currently applied in interstitial PDT of prostate cancer, there is no limitation to follow the same concept and generalize this system for PDT of other internal tumors, such as glioblastoma, where bioresorbable fibers would be highly desirable and better choice than standard non-degradable fibers.

This study establishes the foundation for an interesting perspective in extended interstitial monitoring of PS concentration with implantable bioresorbable fibers and can have positive implications for planning, optimizing, and predicting or monitoring treatment outcomes.^41^ Moreover, the concept of implantable optical fibers and implantation procedures has previously been validated with silica MM fibers in mice for long-term monitoring in optogenetic applications.^34^ Combining DFT with other diffuse optical modalities such as DOS and DCS in the future, for which the bioresorbable fibers have been previously validated,^28^^,^^29^ within a single bioresorbable platform could provide information about the complementary physiological and biochemical signals, which will find significant insights in enhancing the interstitial therapeutic outcomes.

## 5 Conclusion

In this study, we have proposed for the first time the use of bioresorbable optical fibers for DFT in PDT of tumors. By detecting the fluorescent light originating from the PS drug, the spatial distribution of the drug was estimated using the CPG fibers within phantoms with tumor-mimicking inclusions. The results demonstrated good agreement compared with the PDT system equipped with standard silica fibers, in both spherical and ellipsoidal inclusions. The demonstrated concept of monitoring PS distribution in PDT using CPG fibers marks a step forward in the development of an implantable and resorbable multifunctional theranostic platform. Further exploration should focus on extended monitoring with fiber degradation, followed by *in vivo* validation.

## Data Availability

Data underlying the results presented in this paper are not publicly available at this time but may be obtained from the authors upon reasonable request.
